# Baseline testing in cluster randomised controlled trials: should this be done?

**DOI:** 10.1186/s12874-019-0750-8

**Published:** 2019-05-17

**Authors:** Jaime E. Bolzern, Alex Mitchell, David J. Torgerson

**Affiliations:** 1 Hull York Medical School, York, UK; 2 Department of Health Sciences, York Trials Unit, University of York, York, UK

## Abstract

**Background:**

Comparisons of baseline covariates in randomised controlled trials whilst often undertaken is regarded by many as an exercise in futility. Because of randomisation the null hypothesis is true for baseline comparisons and therefore any differences will occur by chance. However, this is only the case if allocations are not known in advance of recruitment. If this occurs then selection bias at randomisation may be present and it is possible that the statistical testing of covariates may unveil selection bias. In this paper we show that this is particularly the case for cluster randomised trials when post-randomised recruitment often occurs and can lead to selection bias.

**Main text:**

We take a recently published cluster randomised trial that has suffered from selection bias due to differential recruitment and calculate baseline *p* values. We show that statistically significant imbalances of *p* < 0.0001 occurred in 5 of the 10 covariates. In comparison for an individually randomised trial that had no evidence of selection bias only 1 *p* value of *p* < 0.05 out of 20 tests was observed. Had baseline *p* values for the cluster trial been presented to journal editors, reviewers and readers then the results of the trial might have been treated with more caution.

**Conclusion:**

We argue that the blanket ban of baseline testing as advocated by some may reduce the chance of identifying deficient cluster randomised trials and this opposition should be reconsidered for cluster trials.

## Background

Randomisation ensures that the groups formed are equivalent in all known and unknown variables except through chance [1]. In reports of randomised controlled trials (RCTs) we often see, alongside the description of the characteristics of trial participants, statistical testing of any imbalance in each characteristic between the randomised groups to ‘check’ that the randomisation has not ‘failed’. There is some debate about the validity of doing this.

### Baseline testing of covariates

One view, which is supported by some leading medical journals (e.g., the *BMJ* and the *Lancet*) and many statisticians [2, 3] and specifically advised against in the CONSORT statement for individually randomised trials [4], is that baseline testing is illogical, irrelevant and, possibly, misleading [5]. The argument for not doing baseline testing is along the following lines: first, assuming robust randomisation we know the null is true: there is no difference between the randomised groups in any of the measured variables except by chance. Second, with multiple statistical tests of 20 or more baseline variables it is almost inevitable that one or two will prove to be ‘statistically significant’. Using significant or non-significant findings of these tests to inform the trial analysis can produce an inefficient analysis. Consider a variable that is highly predictive of outcome (e.g., baseline pain scores). If this variable has a slight (not statistically significant) or zero imbalance we may decide not to include it as a covariate in the final statistical model. Failure to include a powerful predictor, even if it is in balance, will reduce the power of the analysis and if there is an imbalance this will mean the post treatment differences are also biased.

In contrast, one argument for doing baseline testing or at least allowing it to be presented in journal papers (e.g., some papers published in the *Journal of the American Medical Association* or the *New England Journal of Medicine*) is to check that the randomisation has not been subverted [6]. Proponents of this view, whilst accepting in a properly randomised trial that on average one out of every 20 baseline tests will be statistically significant, argue that if there are several variables favouring one group or another or there is a highly statistically significant difference in a key variable then this could be evidence of research misconduct. For example, an RCT of surgery showed statistically significant differences in ages between treated groups and on further investigation it was found that three out of five centres were subverting treatment allocation [7]. In this paper we revisit this debate with respect to cluster randomised controlled trials.

## Main

### Cluster randomised trials

Cluster randomised trials are when participants are randomised as intact groups rather than as individuals. The method is commonly used in educational interventions, where a school or class is the unit of allocation [1], and evaluation of activities, such as health promotion, when doctor might be the unit of allocation, or where there is a risk of the control group being contaminated by the intervention (that is the control participants unintentionally receiving the intervention). Within a cluster trial there are at least two different data levels: cluster and patient. For cluster level variables (e.g., patient list size) then randomisation will on average produce equivalent groups and therefore ‘baseline’ statistical comparisons are subject to the same criticisms or support as for individually randomised trials. However, at the patient level, data can be at increased risk of selection bias compared with individually randomised trials [8]. Often, recruitment to cluster trials occurs after randomisation of the clusters. This reveals the future group allocation to recruiting researchers, clinicians and, sometimes, potential participants. This is akin to publicising the future allocations for an individually randomised trial, something which is now rare but in the past led to biased recruitment [9]. Consequently, there is a significant risk of selective recruitment into cluster trials that introduces selection bias [8]. Currently, journals that do not present baseline testing in individually randomised studies do not present these either for cluster trials. We think that it may be acceptable to present baseline statistical tests for cluster trials that recruit participants *after* randomisation to assess whether there is evidence of selection bias. If such testing were undertaken and this showed a greater than expected number of variables that were statistically significant this may then warrant a more cautious interpretation of the trial’s results.

### Baseline *p* values

The role of p values to assess the equivalence of a trial’s treatment groups is quite different in cluster trials compared with individually randomised studies. In the latter *p* values are linked to the randomisation of the groups and this drives the argument as to whether or not it is valid and helpful to calculate and report them. In contrast for cluster trials, where recruitment has taken place after randomisation, *p* values are no longer entirely linked to the randomisation process, rather they are linked to the recruitment method, which may result in non-random samples being compared. In the following we will examine two case studies: one a cluster randomised trial and the other an individually randomised trial. Both of these were identified from a recent review by Bolzern [10] and colleagues. The aim of these case studies is to demonstrate the distribution of baseline *p* values in a study where there is no evidence of selection bias in an individually randomised trial as recruitment has occurred before randomisation compared with a trial where recruitment has taken place after randomisation, which is often the case in a cluster RCT.

#### Case study: cluster randomised trial

**Fig. 1:**
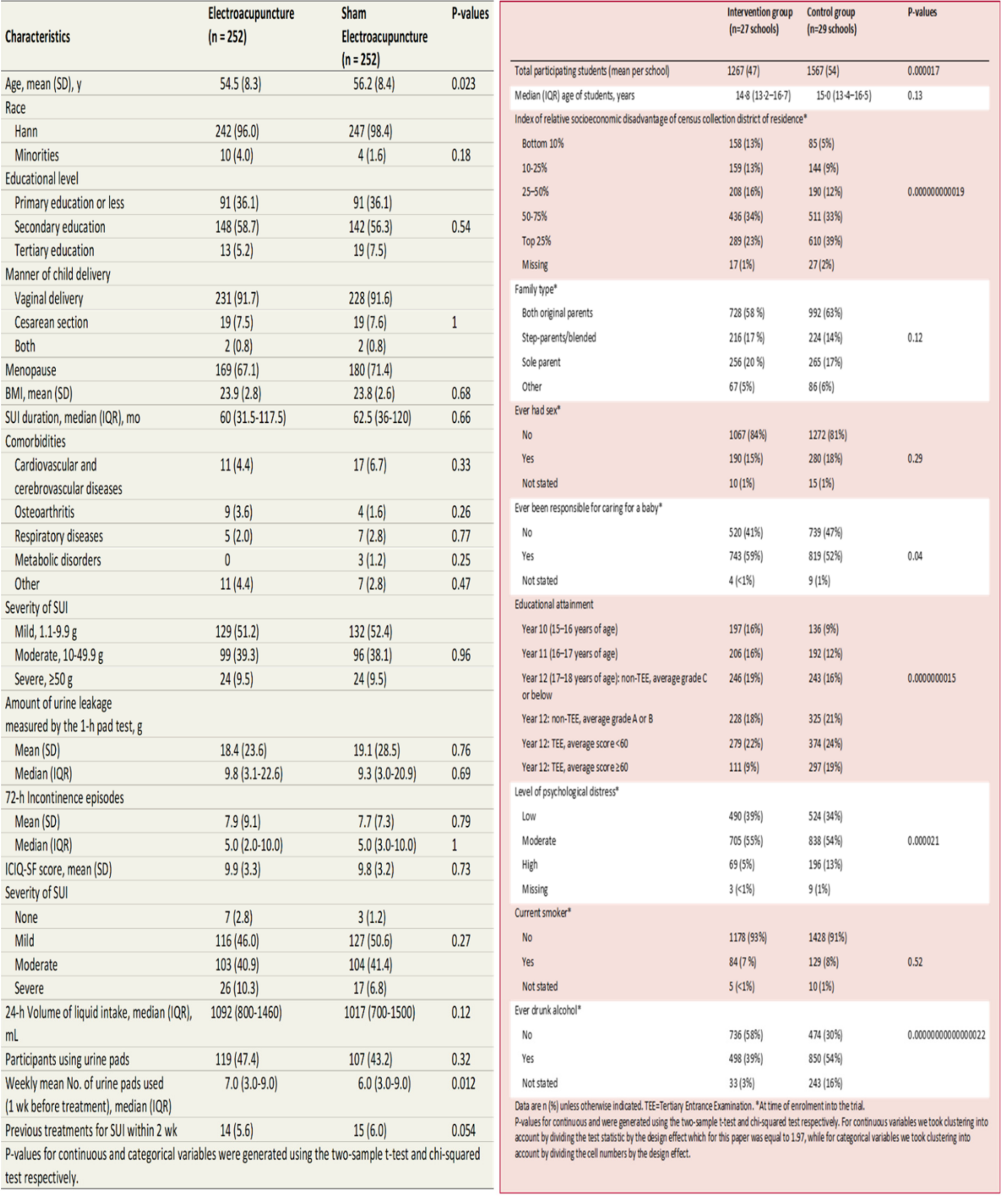
baseline comparison of an individually randomised trial and a cluster randomised trial

The cluster trial by Brinkman et al. [11] evaluated the role of infant simulators to prevent teenage pregnancies. In the trial 57 schools from Western Australia were allocated using simple randomisation, via a table of random numbers, into two groups. After randomisation girls aged 13–15 years were recruited into the trial. Post randomisation recruitment to the study groups was uneven with 50% of eligible girls being recruited in the control schools compared with 58% in the intervention group. Girls in the intervention group as well as receiving the standard health curriculum also received the ‘Virtual Infant Parenting’ programme which consisted of small group teaching with the participants taking a virtual infant home for the weekend. The aim of the intervention was to try and reduce unwanted teenage pregnancies. The results showed that 17% of girls in the intervention group had a pregnancy event compared with 11% of the control group (*p* = 0.00044). The authors noted that the control group had a higher proportion of girls from higher socioeconomic groups, were living with both parents and less likely to have been responsible for caring for a baby. In the original paper no baseline *p* values were presented and we have calculated these and present them in the table. As the Fig. 1 shows of the 10 p values calculated six are statistically significant with five of these being highly significant. The trial design could have easily avoided this problem by simply identifying all of the eligible girls and gaining their consent before randomisation – this, then, would have avoided recruitment bias.

#### Case study: individually randomised trial

The second case study is an individually randomised trial by Liu and colleagues [12]. This study was a RCT to evaluate the role of acupuncture for reducing urine leakage among women with stress urinary incontinence. Women were recruited from this trial from 12 hospitals in China. Block randomisation (size 6) was used to stratify by hospital and 504 women were randomised to either ‘true’ or ‘sham’ acupuncture. The trial found a significant benefit of true acupuncture on symptoms (*p* < 0.001). In the Fig. 1 we have calculated baseline *p* values and with over 20 calculations only one (age) was marginally statistically significant (*p* = 0.023); thus suggesting selection bias in this trial was unlikely.

### Estimation of baseline *p* values

In our analysis of p values for the two case studies we used the aggregate data. The *p*-values for the study by Liu et al. were calculated by using the two-sample t-test for continuous variables and the chi-squared test for categorical variables. The p-values for the study by Brinkman et al. were calculated using a similar method to that used for Liu et al., with an adjustment made for clustering. For continuous variables clustering was taken into account by dividing the test statistic by the design effect, while for categorical variables clustering was taken into account by dividing the cell numbers by the design effect, which for the study by Brinkman et al. was equal to 1.97. The design effect was calculated using the ICC assumed in the sample size calculation, the number of clusters randomised and the total number of participants who consented and participated. As the authors only had access to the information provided in the original baseline table, this was considered the best method available. However, the authors recommend that statisticians with access to individual participant data calculate baseline *p*-values adjusted for clustering by fitting a mixed effects model for each baseline covariate in the table, adjusting for the treatment group as a fixed effect and cluster as a random effect.

## Conclusion

Recruitment bias, which is simply a type of post randomisation selection bias, is a major problem for significant numbers of cluster randomised trials. It is important that the reader of a cluster trial can identify whether such a problem exists. In this paper we are recommending that, for cluster trials, formally testing for baseline imbalances of patient level data should be considered to help identify weak cluster trials. We have shown in two case studies the distribution of *p* values that might occur when there is clear evidence of recruitment bias (Brinkman et al.^11^) compared with a study when recruitment bias is unlikely. If baseline p values had been presented in the Brinkman trial it may have led to a more nuanced interpretation of the results and perhaps the findings would not be deemed as credible by such as the United Kingdom’s Health service: NHS choices, which concluded that: “*This trial had a good study design*” [13].

We are aware of that ‘baseline statistical tests’ are seen as an anathema, to some, in individually randomised trials. However, the arguments over baseline testing using *p* values for individually randomised trials are not relevant here. We are not testing whether the randomisation process ‘worked’. We are trying, instead, to identify any evidence that suggests that recruitment bias has occurred and the pre-intervention group averages are not equivalent. One of this paper’s referees pointed out that covariate imbalances can be identified by study statisticians using the ‘eyeball’ test negating the need for the use of *p* values. For experienced trialists baseline imbalances can be identified without the use of *p* values; however, most readers of trial research are not experienced statisticians or trial methodologists. We believe placing p values alongside these differences makes them difficult to ignore and furthermore, non-technical experts, such as staff at NHS choices, might not have rated that the trial had a ‘good study design’.

We believe the use of *p* values is one tool that can help identify recruitment bias. Note that in so doing we are not checking that the randomisation procedure has ‘worked’: we assume this to be the case. Using *p* values in this way is in addition to the quality assessment of the likelihood of this occurring through the implementation of the cluster design. Indeed, the use of baseline p values could be easily combined with other quality assurance measures such as the Timeline cluster approach, which graphically describes the recruitment process and draws the reader’s attention to whether or not recruitment had occurred before or after randomisation [14].

The problem of selection bias, due to recruitment bias, in cluster trials has been identified as a major problem for a number of years [8, 15]. The recommendation to identify and recruit participants before randomisation, if possible, has not been implemented in many trials. Indeed, a recent review of 23 cluster RCTs published between 2015 and 2017 found only four (17%) recruited participants before randomisation [10]. Whilst the recommendation of recruitment before randomisation, if possible, should remain, perhaps CONSORT guidance for cluster trials should include the suggestion of baseline testing of patient level data, as it is currently silent on this issue. Including baseline *p* values for cluster trials may result in better cluster trial designs in the future. Using statistical significance testing on baseline patient level variables in cluster randomised trials, that recruit participants after randomisation, should be encouraged as this is a relatively easy method to detect potential selection bias. This should lead to a more cautious interpretation of cluster trials where there is a high prevalence of statistically significant *p* values among patient level co-variates.

## Abbreviations

CONSORT: Consolidated Standard of Reporting Trials
NHS: National Health Service
RCT: randomised controlled trial

## References

[1] Torgerson DJ, Torgerson CJ (2008). Designing randomised trials in health, education and the social sciences: an introduction.

[2] Assman SF, Pocock SJ, Enos LE, Kasten LE (2000). Subgroup analysis and other (mis)uses of baseline data in clinical trials. Lancet.

[3] Senn S (1994). Testing for baseline balance in clinical trials. Stats Medicine.

[4] Moher D, Hopewell S, Schulz KF, Montori V, Gotzsche PC, Devereaux PJ, Elbourne D, Egger M, Altman DG (2010). CONSORT 2010 explanation and elaboration: updated guidelines for reporting parallel group randomised trials. BMJ.

[5] Altman DG, Armitage P, Colton T (2005). Adjustment for covariate imbalance. Encyclopedia of biostatistics.

[6] Berger V (2005). Selection bias and covariate imbalances in randomized clinical trials statistics in practice.

[7] Kennedy ADM, Torgerson DJ, Campbell MK, Grant AM (2017). Subversion of allocation concealment in a randomised controlled trial: a historical case study. Trials.

[8] Puffer S, Torgerson DJ, Watson J (2003). Evidence for risk of Bias in cluster randomised trials: a review of recent trials published in three general medical journals. BMJ.

[9] Schulz KF (1995). Subverting randomization in controlled trials. JAMA.

[10] Bolzern J, Mnyama N, Bosanquet K, Torgerson DJ (2018). A review of cluster randomized trials found statistical evidence of selection bias. J Clin Epidemiol.

[11] Brinkman SA, Johnson SE, Codde JP, Hart MB (2016). Efficacy of infant simulator programmes to prevent teenage pregnancy: a school-based cluster randomised controlled trial in Western Australia. Lancet.

[12] Liu Z, Liu Y, Huanfang X, He L, Chen Y (2017). Effect of electroacupuncture on urinary leakage among women with stress incontinence: a randomized clinical trial. JAMA.

[13] NHS choices accessed 24th July 2018. https://www.nhs.uk/news/pregnancy-and-child/baby-doll-simulators-may-actually-increase-teen-pregnancy-rates/. Accessed 24 July 2018.

[14] Caille A, Kerry S, Tavernier E, Leyrat C, Eldridge S, Giraudeau B (2016). Timeline cluster: a graphical tool to identify risk of bias in cluster randomised trials. BMJ.

[15] Torgerson DJ (2001). Contamination in trials: is cluster randomisation the answer?. BMJ.

